# A cross-sectional study of physical activity behaviour and associations with wellbeing during the UK coronavirus lockdown

**DOI:** 10.1177/1359105321999710

**Published:** 2021-03-03

**Authors:** Carly J Wood, Jo Barton, Nina Smyth

**Affiliations:** 1 University of Essex, UK; 2 University of Westminster, UK

**Keywords:** anxiety, depression, exercise behaviour, physical activity, stress, well-being

## Abstract

This study assessed physical activity (PA) and wellbeing during lockdown. UK adults reported their PA in the previous week, perception of PA importance (more, less, same) and wellbeing, depression, anxiety and stress. One-way ANOVA compared PA and wellbeing by PA importance. The ‘less’ importance group did less PA than the ‘more’ and ‘same’ (*p* < 0.05) importance group; and scored worse on all wellbeing measures than the ‘same’ importance group (*p* < 0.01). They also had worse wellbeing, depression and anxiety than the ‘more’ importance group (*p* < 0.05). Strategies to overcome the impact of the pandemic should aim to increase PA.

## Introduction

To reduce the spread of coronavirus, the majority of Governments imposed restrictions on movement and recommended that the population should stay in their homes. In the UK, these first lockdown restrictions were implemented in March 2020. There is no doubt that the pandemic has had a significant impact on our way of life. If we consider this in the context of Maslow’s (1943) statement of human needs, which assumes a hierarchy of seven overlapping needs that are key to homeostasis; there are several key needs upon which the pandemic has a negative effect. In terms of physiological needs, the pandemic is likely to have caused disturbances to sleep and increases in comfort eating, resulting in reduced weight control (Matias et al., 2020). The pandemic has also prevented individuals from affiliating with others; and is likely to have negatively affected feelings of self-esteem and status due to potential job losses and financial struggles (Matias et al., 2020). The disruption of all these needs causes imbalances in homeostasis and subsequent negative health outcomes if homeostasis is not restored. In fact, recent evidence suggests that in similar pandemics where quarantine has been imposed, there has been increased prevalence of symptoms of post-traumatic stress disorder and feelings of loneliness, depression and anger (Brooks et al., 2020).

There is also evidence of the direct negative effect of the coronavirus lockdown. Evidence from a largescale longitudinal study of households in the UK (Understanding Society) revealed that average General Health Questionnaire (GHQ) scores in April 2020, reflecting levels of psychiatric distress, were 1.23 points (equivalent to 10.8%) higher than scores between January 2017 and May 2019; and 0.94 points (equivalent to 8.1%) higher than estimated values for April 2020 based on predictive modelling (Banks and Xu, 2020). A one-point difference in GHQ scores has previously been found to reflect differences between the top and bottom quintiles for household income; with the prior 4 years of the study only documenting a half point rise in average scores (Banks and Xu, 2020). Indeed, this half point rise was enough to raise concern over the increasing prevalence of mental ill health. Further data by Chandola et al. (2020) revealed that 30% of individuals without a common mental health disorder such as anxiety or depression in 2017–2019 reported having a common mental health disorder in April 2020; with an 8.4% increase in mental distress in April 2020 compared to 2018–2019 (Pierce et al., 2020).

To cope with the physiological and psychological impact of the pandemic and to restore homeostasis; self-care has been highlighted as increasingly important (Matias et al., 2020). Physical activity (PA), a form of self-care, has consistently been demonstrated to improve both physical and mental health (Biddle et al., 2019; Reiner et al., 2013; Saxena et al., 2005; Warburton, 2006; Warburton and Bredin, 2017). In the context of key physiological and psychological needs that are disrupted by the pandemic (Maslow, 1943; Matias et al., 2020); PA has previously been shown to improve sleep (Flausino et al., 2012; Reid et al., 2010; Yang et al., 2012), aid weight control (Petridou et al., 2019); and enhance self-esteem due to feelings of control, competence and achievement (Opdenacker et al., 2009; Standage and Ryan, 2012; Sani et al., 2016). In addition, the ability to access online workouts during the pandemic has also provided individuals with the ability to affiliate with others and build social connections (Matias et al., 2020).

Despite the potential importance of PA for ameliorating the negative impacts of the pandemic; a large survey of the impact of the pandemic on the psychological and social experiences of adults in UK revealed that 1 week into the lockdown restrictions, 85.9% of respondents (*n* = 19,393) did not engage in any moderate physical activity (MPA) on the previous weekday. Approximately 25% also reported not engaging in any activity at all, both 1 and 5 weeks into the lockdown restrictions (Fancourt et al., 2020a, 2020b). Although this survey had a high response rate, it only asked participants about their PA on 1 weekday; and nearly half of respondents had a long-term physical health condition which could have influenced their PA participation. Surveys conducted in France and Switzerland found that sedentary time, walking and MPA significantly increased during lockdown, while vigorous physical activity (VPA) significantly decreased (Cheval et al., 2020). However, it is not clear how this compares to the UK, particularly given slightly lower rates of PA prior to the coronavirus (World Health Organisation (WHO), 2018); and PA levels were recalled retrospectively which is likely to have provided biased results. Furthermore, none of these surveys considered the influence of an individual’s perception of the importance of PA during the pandemic on PA levels. If PA is a key strategy for allowing an individual to cope with, and ameliorate the negative effects of the pandemic; it is likely that their PA patterns might differ to those who are not using PA as a coping mechanism.

The aims of this study were therefore:

i. To examine the impact of the UK lockdown restrictions on PA behaviours;
ii. To determine whether PA levels during lockdown differed according to participants perception of the importance of PA;
iii. To determine whether wellbeing differed according to participants PA levels during lockdown;
iv. To determine which factors were the strongest predictors of PA and wellbeing during lockdown.

## Methodology

### Participants and procedures

Participants were recruited via various routes. First, the study was advertised on social media, including Facebook and twitter. A snowballing sampling strategy was used to recruit participants; whilst details of the study were also shared amongst the researchers own social media groups. The study was also advertised on institutional and research group websites; and shared with the researcher’s colleagues, collaborators and contacts, all of whom were asked to share the survey with their networks. All participants were aged 18 years+ and residing in the UK at the time of survey completion. Participants completed the survey between 1st May and 2nd June 2020 during the UK Government’s Coronavirus restrictions. In line with these restrictions’ individuals could only leave their homes for the following reasons: (i) to shop for necessities, for example food and medicine; (ii) for one form of exercise a day, alone or with household members; (iii) for any medical need, or to provide care or to help a vulnerable person and; (iv) to travel for work purposes, if the work could not be conducted from home. From the 13th May these restrictions were eased slightly to allow unlimited exercise and time outdoors (GOV.UK, 2020).

All participants completed the survey electronically via Qualtrics and provided their consent to take part in the study. Ethical approval was granted by the School of Sport, Rehabilitation and Exercise Sciences Ethics Sub-committee at the University of Essex. The final sample consisted of 315 participants, including 77 males and 237 females (one participant did not reveal their gender). All submitted responses were included in the analysis.

### Measures

#### Demographic and lifestyle information

Participants were asked to provide a range of demographic data including age, gender and ethnicity. They were also asked to rate their socioeconomic status (SES) on a scale from one to ten, with one representing the people who are worst off and have the least money, education and worst jobs and ten representing those who are best off, with the most money, education and best jobs. Participants also detailed their employment status prior to the pandemic and where they were performing their work during the pandemic, including whether they were classified as a key worker or furloughed. Key workers are defined as individuals whose work is critical to the coronavirus response and to keeping the country running, for example those in health and social care, education, food and key public services (GOV.UK, 2021a). Participants were also asked to identify whether they had access to a private or shared garden and whether they were asked to stay indoors (‘shield’) by the National Health Service (NHS). Individuals in the UK were asked to shield if there were defined as clinically extremely vulnerable and were therefore at very high risk of severe illness from coronavirus (GOV.UK, 2021b). This included individuals with severe respiratory conditions, specific cancers and on immunosuppression therapies.

#### Physical activity

Participants completed the International Physical Activity Questionnaire Short Form (IPAQ-SF; Craig et al., 2003); which asks about vigorous and moderate PA, and walking activities in the last 7 days and the time spent in each intensity of PA. The metabolic equivalent (MET) minutes for each PA intensity were calculated using the following equations:

$$\begin{array}{l} \mathrm{Vigorous}\;\mathrm{PA}\;\left(\mathrm{VPA}\right)\;\mathrm{MET}\;\mathrm{minutes}=8.0\;X\;VPA\;days\;X\;VPA\;minutes\\ \mathrm{Moderate}\;\mathrm{PA}\;\left(\mathrm{MPA}\right)\;\mathrm{MET}\;\mathrm{minutes}=4.0\;X\;MPA\;days\;X\;MPA\;minutes\\ \mathrm{Walking}\;\mathrm{MET}\;\mathrm{Minutes}=\;3.3\;X\;walking\;days\;X\;walking\;minutes \end{array}$$

The total MET minutes was then determined by summing VPA, MPA and walking MET minutes. Participants overall PA during the pandemic was also categorised as either ‘low’, ‘moderate’ or ‘high’ (entitled PA level category). Individuals were categorised as highly active if they performed VPA on at least 3 days per week accumulating at least 1500 MET-minutes per week; or performed five or more days of any combination of walking, MPA or VPA achieving a minimum of 3000MET-minutes/week. Individuals were classified as moderately active if they performed three or more days of VPA for at least 20 minutes, five or more days of MPA or walking for at least 30 minutes or five or more days of any combination of walking, MPA or VPA achieving a minimum of 600MET-minutes/week. Individuals not meeting the criteria for high or moderately active were classified as low active (IPAQ Research Committee, 2005).

Participants also reported the time spent sitting on an average weekday prior to the pandemic and identified their main modes and locations of PA (up to a maximum of three) both before and during the pandemic. Finally, participants were asked to indicate how important physical activity was during the coronavirus lockdown compared to before the pandemic; selecting whether it was more important, of the same importance or of less importance.

#### Warwick Edinburgh Mental Wellbeing Scale Short Form

Participants wellbeing in the last month was assessed via the short form Warwick Edinburgh Mental Wellbeing Scale (SWEMWBS; Fat et al., 2017; Tennant et al., 2007). The SWEMWBS consists of seven positively worded items from the full 14-item scale and is scored by summing responses to each item, which are scored on a five-point Likert scale from 1 (none of the time) to 5 (all of the time). The raw SWEMWBS scores were converted to metric scores (Stewart-Brown et al., 2009) prior to data analyses; with scores ranging from 7 to 35 and higher scores indicating better wellbeing. The SWEMWBS has been reported to have a Cronbach’s alpha of 0.84 using England population-level data (Fat et al., 2017); with correlations between the full and short version being 0.954 (Stewart-Brown et al., 2009). In the current sample the Cronbach’s alpha was 0.82, indicating very good reliability.

#### Depression, anxiety and stress

The Depression, Anxiety and Stress Scale-21 is a 21-item scale that is designed to measure the emotional states of depression, anxiety and stress (Henry and Crawford, 2005). Each of these sub-scales contain seven items with responses categories from 0 (did not apply to me at all) to 3 (applied to me very much or most of the time). The overall score for each sub-scale is calculated by summing the items and multiplying by 2; with scores ranging from 0 to 42 and a higher score representing greater feelings of depression, anxiety or stress. The sub-scales have previously been demonstrated to have reliabilities of 0.88, 0.82 and 0.90 respectively (Henry and Crawford, 2005). In the current sample the subscales had Cronbach’s alphas of 0.89, 0.80 and 0.88 respectively, indicating very good reliability.

### Statistical analyses

One way between subjects ANOVA with Tukey post hoc comparisons was used to compare PA variables during lockdown (VPA MET-minutes, MPA MET-minutes, walking MET-minutes, total PA MET-minutes, time sitting) by participants perception of the importance of PA during lockdown (more important, of the same importance, less important). Multiple regression analysis was also conducted to examine the impact of garden access, employment status during the pandemic, shielding status and PA importance on total PA MET-minutes and sitting time during lockdown. The variables of age, gender and SES were entered into block one of the regression model to allow the effects of these variables to be controlled for.

A one sample *t*-test was conducted to compare the SWEMWBS score for the sample to the mean population score according to the Fat et al. (2017). One way between ANOVA with post hoc Tukey was used to compare the effect of PA level category (low, moderate, high) and PA importance (more, same or less) during lockdown on wellbeing variables (SWEMWBS, depression, anxiety and stress). Multiple regression was also conducted for the wellbeing variables. The model explored the impact of garden access, employment status during the pandemic, shielding status, PA importance and total PA during on each of the wellbeing variables, whilst controlling for the effects of age, gender and SES.

## Results

### Participants

Table 1 displays the demographic details for the participants. The average age of the participants was 40.2 ± 13.5years, with an average socioeconomic status of 6.7 ± 1.3 (ranging from 3 to 10). Most participants were female and of a white background. The majority were in full time employment prior to the pandemic and continued to work during the pandemic; however, 15.0% were furloughed and 22.9% were key workers. Most participants reported that they had a private garden (84.8%). Some participants were also shielding (11.4%).

**Table 1.** Demographic and lifestyle details for participants.

	Current Sample
	*N* (%)
Gender	
Male	77 (24.4)
Female	237 (75.2)
Ethnicity	
White background	303 (96.2)
Asian background	4 (1.3)
Mixed background	8 (2.5)
Employment	
Full time	171 (54.3)
Part time	50 (15.9)
Self-employed	30 (9.5)
Retired	29 (9.2)
Other	34 (10.8)
Employment status during the pandemic	
Furloughed	47 (15.0)
Working (all except keyworkers)	133 (42.4)
Key worker	72 (22.9)
Not applicable (unemployed/retired, etc.)	62 (19.7)
Shielding	
Yes	36 (11.4)
No	278 (88.3)
Garden	
Private garden	267 (84.8)
Shared garden	17 (5.4)
No garden	30 (9.5)

### Physical activity during lockdown

Table 2 displays changes in PA variables from pre- to during- lockdown. Prior to the pandemic the most frequent modes of PA reported were outdoor walking, gym-based activities and fitness classes; with participants mostly working out in a gym/leisure centre or local park/recreation space. During lockdown, outdoor walking remained the most common mode of PA, followed by outdoor running and online fitness classes; whilst most participants worked out at home or in a local park/recreation space. Most participants were moderately active during the lockdown (51.6%); with 31.0% being classed as highly active and 17.4% having low levels of PA. More than half (54.3%; *n* = 171) of participants reported that exercise was more important during lockdown than it was prior to the lockdown; with 8.3% (*n* = 26) reporting that it was less important and 37.5% (*n* = 118) indicating no change in importance.

**Table 2.** Physical activity modes and locations pre- and during- the coronavirus lockdown.

	Pre-pandemic (%)	During lockdown (%)	Change (+/−) (%)
Physical activity mode			
Gym-based	40.0	–	–
Outdoor walking	60.0	76.2	15.9 (+)
Outdoor running	23.2	33.3	10.3 (+)
Outdoor cycling	7.3	16.2	9.2 (+)
Sports	18.4	–	–
Fitness classes (online during pandemic)	23.8	33.3	8.6 (+)
Gardening/conservation	14.3	27.3	13.0 (+)
Other (e.g. outdoor swimming)	14.0	17.5	3.5 (+)
Physical activity location			
Indoors at home	16.8	51.4	34.3 (+)
Indoors at gym/leisure centre	45.7	–	–
Outdoors in an urban area	24.8	26.7	2.9 (+)
Outdoors in garden	16.5	41.9	25.4 (+)
Outdoors at local park/recreation space	36.8	45.4	8.6 (+)
Outdoors at an allotment	1.3	1.6	0.3 (+)
Outdoors in a natural environment (e.g. countryside, woodland)	31.4	27.3	4.1 (−)

**Figure 1.**
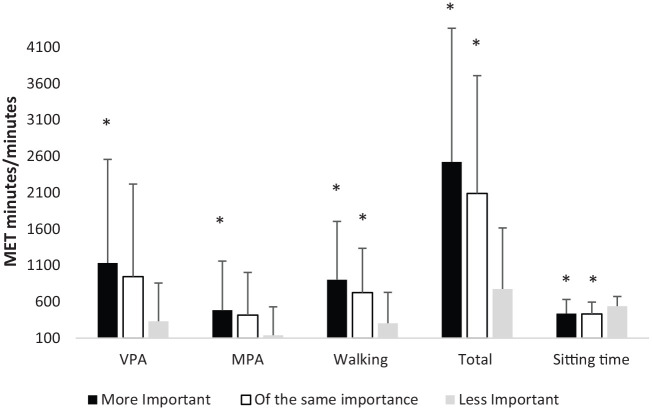
Metabolic equivalent minutes of physical activity and time spent sitting during lockdown by physical activity importance. *Indicates a significant difference to the less important group (*p* < 0.005).

One way between subjects ANOVA revealed a significant effect of PA importance during lockdown on VPA MET minutes [*F*(2,298) = 4.284; *p* = 0.015; η^2^ = 0.028], MPA MET minutes [*F*(2,298) = 3.475; *p* = 0.032; *η*^2^ = 0.023], walking MET minutes [*F*(2,298) = 8.635; *p* < 0.001; η^2^ = 0.055], total PA MET minutes [*F*(2,298) = 12.34; *p* < 0.001; η^2^ = 0.077] and sitting time [*F*(2.297) = 3.779; *p* = 0.024; η^2^ = 0.025]. Participants who reported that PA was ‘more important’ participated in more PA, at all intensities (Figure 1). Tukey post hoc comparisons revealed that participants perceiving PA to be ‘more important’ engaged in significantly more PA MET minutes at all intensities; and less sitting than those who perceived it to be less important (VPA *p* = 0.012, MPA *p* = 0.025; walking *p* < 0.001; Total *p* < 0.001; sitting *p* = 0.026). The ‘same importance’ group also engaged in significantly more walking MET minutes (*p* = 0.018), total MET minutes (*p* < 0.001) and less sitting (*p* = 0.021) than the ‘less important’ group.

### Physical activity and wellbeing during lockdown

The mean SWEMWBS score for the overall sample was 21.5 ± 3.5. A one samples t-test revealed that this was significantly lower than the mean population score reported by Fat et al. (2017) of 23.7 ± 3.9 (*t*(314) = −11.45; *p* < 0.001). The mean sample score for depression, anxiety and stress were 10.2 ± 8.2, 5.4 ± 6.4 and 13.3 ± 8.4 respectively, representing ‘mild’ depression and ‘normal’ anxiety and stress scores (Henry and Crawford, 2005).

Based on their reported PA, participants were classified as ‘low’ active, ‘moderately’ active or ‘highly’ active. There was a significant effect of PA level during lockdown on scores for wellbeing [*F*(2,284) = 6.197; *p* = 0.002; η^2^ = 0.042], depression [*F*(2,284) = 10.755; *p* < 0.001; η^2^ = 0.070], anxiety [*F*(2,283) = 9.890; *p* < 0.001; η^2^ = 0.065] and stress [*F*(2,284) = 5.360; *p* = 0.005; η^2^ = 0.036]. Tukey Post hoc comparisons revealed that those who had ‘low’ levels of PA had significantly worse scores on all wellbeing measures than both the ‘moderately active’ (wellbeing *p* = 0.004; depression *p* < 0.001; anxiety *p* < 0.001; stress *p* = 0.032) and the ‘highly’ active group (wellbeing *p* = 004; depression *p* < 0.001; anxiety *p* < 0.001; stress *p* = 0.004; Table 3). There were no significant differences between the moderately active and highly active groups (*p* > 0.05).

**Table 3.** Mean (SD) wellbeing scores according to physical activity level and importance.

	SWEMWBS	Depression	Anxiety	Stress
PA category				
Low (*n* = 50)	19.9 (4.5)	14.8 (11.1)	8.8 (9.0)	16.4 (10.2)
Moderate (*n* = 148)	21.7 (3.0)^a^	9.5 (6.9)^a^	4.8 (5.5)^a^	13.0 (8.1)^a^
High (*n* = 89)	21.9 (3.4)^a^	8.7 (7.1)^a^	4.2 (5.3)^a^	11.7 (6.8)^a^
PA importance				
Less important (*n* = 26)	19.6 (4.3)	15.9 (10.7)	9.5 (9.7)	17.3 (10.6)
Same importance (*n* = 118)	21.8 (3.6)^b^	9.0 (8.0)^b^	3.9 (4.4)^b^	11.7 (7.6)^b^
More important (*n* = 171)	21.5 (3.2)^b^	10.2 (7.6)^b^	5.8 (6.9)^b^	13.8 (8.3)

a Indicates significant different to low active group.

b Indicates a indicates significant different to those rating PA as less important.

There was a significant effect of perceived importance of PA during lockdown on well-being [*F*(2,312) = 4.528; *p* = 0.012; η^2^ = 0.028], depression [*F*(2,312) = 7.869; *p* < 0.001; η^2^ = 0.048], anxiety [*F*(2,311) = 9.289; *p* < 0.001; η^2^ = 0.056] and stress [*F*(2,312) = 5.608; *p* = 0.004; η^2^ = 0.035]. Tukey post hoc comparisons revealed that those who reported that PA was less important scored significantly worse on all wellbeing measures (wellbeing *p* = 0.008; depression *p* < 0.001; anxiety *p* < 0.001; stress *p* = 0.005) than those who reported that PA was of the same importance. Those who reported that PA was less important also had significantly lower wellbeing (*p* = 0.023), more depression (*p* = 0.002) and more anxiety (*p* = 0.014) than those who said PA was more important. Those who said PA was more important had greater anxiety than those who said it was of the same importance (*p* = 0.033; Table 3). There were no significant differences in wellbeing, depression or stress between the same importance and more importance groups (*p* > 0.05).

### Predictors of physical activity and wellbeing during lockdown

Table 4 displays the regression models for total PA MET minutes, sitting time and wellbeing variables. Hierarchical multiple regression revealed that the overall model significantly predicted 7.5% of the variance in total PA MET minutes [*F*(7,298) = 4.891; *p* < 0.001] and 3.4% of the variance in sitting time [*F*(7,298) = 2.47; *p* = 0.018]. After controlling for the effect of age, gender and SES the change in the variance remained significant (see Table 4); with greater PA importance during lockdown (*p* < 0.001) and older age (p = 0.046) significantly predicting greater total PA during lockdown; and having access to a garden (p = 0.028) and older age (p = 0.047) significantly predicting less sitting time (*p* = 0.028).

**Table 4.** Regression models for physical activity and wellbeing variables during lockdown.

	*R*^2^ change	Significant of *F* change	Age	Gender	SES	Working	Garden access	Shielding status	Total PA MET minutes	PA importance
Total PA MET minutes	0.075	*F* change (4291) = 6.116; *p* = 0.000	0.114*	−0.094	0.072	−0.024	−0.072	−0.058	–	−0.258*
Sitting time	0.034	*F* change (4291) = 2.652; *p* = 0.033	−0.116*	−0.048	0.020	−0.027	0.127*	−0.094	–	0.104
Wellbeing	0.020	*F* change (5290) = 0.1.34; *p* = 0.250	0.167*	−0.050	251*	0.070	−0.004	0.049	0.083	−0.060
Depression	0.038	*F* change (5290) = 2.55; *p* = 0.028	−0.135*	0.063	−0.228*	−0.104	−0.036	−0.074	−0.117*	0.065
Anxiety	0.026	*F* change (5290) = 1.181; *p* = 0.111	−0.241*	0.031	−0.208*	−0.048	−0.028	−0.097	−0.124*	0.003
Stress	0.015	*F* change (5290) = 1.06; *p* = 0.382	−0.310*	0.082	−0.158*	−.067	−0.011	0.009	−0.109	−0.009

Note. *R*^2^ change represents the variance explained by the independent variables after controlling for the effects of age, gender and socioeconomic status; significance of *F* change indicates the effect of the model after the independent variables have been controlled for.* indicates that the variable significantly predicts the physical activity or wellbeing variable

For the wellbeing variables, the overall model significantly explained 2.0% for the variance in wellbeing scores [*F*(8,298) = 6.077; *p* < 0.001], 3.8% of the variance in depression scores [*F*(8,298) = 5.918; *p* < 0.001], 2.6% of the variance in anxiety scores [*F*(8,298) = 7.409; *p* < 0.001] and 1.5% of the variance in stress scores [*F*(8,298) = 7.619; *p* < 0.001]. After controlling for the effect of age, gender and SES the change in the variance was only significant for depression, where older age (*p* = 0.017), higher SES (p ⩽ 001) and engaging in more PA (*p* = 0.043) predicted lower depression. Older age also significantly predicted better wellbeing (*p* = 0.003) and less anxiety (*p* < 0.001) and stress (*p* < 0.001); whilst higher SES was associated with better wellbeing (*p* < 0.001) as well as lower anxiety (*p* < 0.001) and stress (*p* = 0.004). Engaging in more PA also significantly predicted lower anxiety (*p* = 0.029)

## Discussion

The primary aim of this study was to examine the impact of the coronavirus lockdown on PA behaviours and to determine whether PA levels during lockdown differed according to participants perception of the importance of PA. During lockdown the percentage of participants performing PA at home and in their own gardens increased, likely due to the closure of facilities typically used for PA such as gyms and leisure centres. Both before and during the pandemic walking was reported to be the most common mode of PA, with a greater percentage of respondents walking as a main mode of exercise during the pandemic. There was also an increase in the percentage of participants reporting undertaking gardening, in line with greater garden use for PA.

The perceived importance of PA during the lockdown impacted both PA and sitting time. Those who said PA was more important performed significantly more VPA, MPA, walking and total PA and less sitting than those who said PA was less important; whilst those who said PA was of the same importance also did significantly more walking and total PA, and less sitting than those who said PA was less important. Although not significant, those who reported that PA was more important took part in more VPA, MPA, walking and total PA than those who said that PA was of the same importance. This is supported by the findings of the regression analyses which revealed that PA importance was a unique and significant predictor of total PA. In the context of coronavirus pandemic and the potential disruptions to health (Matias et al., 2020); these findings might suggest that individuals who were using PA as a method of coping with the physiological and psychological effects of the pandemic, placed more importance upon it and were therefore more physically active. It might also be the case that those who rated PA as the same importance already believed it to be important and thus continued to engage in PA, which also helped them to manage their wellbeing.

The idea of attitudes towards PA influencing behavioural outcomes is not new (Chevance et al., 2019; Poobalan et al., 2012); however, beyond the pandemic it would be interesting to determine whether the changes in importance of PA were maintained and whether this had any long-term impact on PA levels. It is suggested that conducting a behaviour for 1 hour per day for 50 days, or half an hour per day for 100 days (approximately 3 months) can produce changes in the brain that result in fixed behaviour changes (Pretty et al., 2017). If changes in participants perception of PA importance resulted in changes in PA throughout the duration of the initial lockdown, which itself was almost 3 months, it is feasible that long term shifts in PA might have occurred. Furthermore, the prolonged duration of the pandemic and the implementation of subsequent lockdown periods might also have contributed to longer term changes in PA. It would also be interesting for future research to explore the reasons for the changes in importance of PA, as this information could help to inform interventions and public health policies.

The secondary aim of this study was to explore whether wellbeing differed according to participants PA levels during lockdown. Overall, the mean score on the SWEMWBS was significantly lower than the UK population norm (Fat et al., 2017); whilst the mean score for depression indicated a ‘mild’ level of depression amongst the sample. These findings support numerous studies documenting the adverse health impacts of this and indeed other similar pandemics (Banks and Xu, 2020; Brooks et al., 2020; Chandola et al., 2020).

Those classified as having ‘low’ activity levels during lockdown experienced significantly worse wellbeing, depression, anxiety and stress than those classified as ‘highly’ or ‘moderately’ active. Furthermore, those individuals who said that PA was less important during lockdown had significantly worse wellbeing, depression, anxiety and stress than those who indicated no change in importance; and worse depression, stress and anxiety than those who indicated that PA was more important. In addition, total PA during the pandemic was a unique and significant predictor of depression and anxiety. In the current study those who were ‘low’ active or reported that PA was less important during the lockdown had scores reflecting ‘moderate’ depression and ‘mild’ stress and anxiety (Henry and Crawford, 2005). These findings further support the growing body of evidence demonstrating links between PA and mental wellbeing (Biddle, 2016; Dore et al., 2018); and are in line with evidence indicating that increased time spent in PA during the coronavirus lockdown was associated with improved mental wellbeing via reductions in depression and anxiety and improved life satisfaction (Bu et al., 2020).

In the context of Maslow’s (1943) statement of human needs, the findings indicate that the effects of the pandemic might have been worse for those who engaged in less PA; with PA playing a role in ameliorating the threats of the pandemic to mental wellbeing. However, these findings must be interpreted with caution as it is not possible to confirm the direction of cause and effect (i.e whether low PA/reduced PA importance led to increased depression, or whether increased depression resulted in low levels of PA/reduced PA importance); or the role of prior levels of wellbeing and external factors such as job and familial losses in this relationship. For example, there are unique predictors of PA in individuals with poor mental wellbeing such as altered adherence and behaviour change, which might influence PA-wellbeing relationships (Rebar and Taylor, 2017).

Although only significant for anxiety, those who said that PA during lockdown was of the same importance had more favourable scores for all wellbeing measures, than those who reported that it was more important. This finding could be explained by individuals who rated PA as more important doing so to directly manage the adverse mental health impacts associated with the coronavirus, or to manage an existing mental health issue for which they could not receive their usual treatment. Although speculation and requiring investigation; this further supports the importance of PA for management, prevention and treatment of mental ill-health (Biddle et al., 2019; Saxena et al., 2005).

Age and SES were associated with wellbeing during lockdown, with younger age and lower SES significantly predicting worse wellbeing, and more depression, anxiety and stress. There is growing evidence to indicate that low SES is linked to poorer physical and mental health across the lifespan, and reduced life expectancy (Hudson, 2010; Kivimaki et al., 2020; Marmot et al., 2020). Furthermore, the coronavirus pandemic has further emphasised these health inequalities; with the most deprived groups often having the most adverse outcomes from catching coronavirus and the restrictions imposed by the government (Bibby et al., 2020). Early evidence also indicates the most adverse mental health impacts of the pandemic have occurred in younger adults (Banks and Xu, 2020; Pierce et al., 2020); perhaps because they are at a stage of their lives where their careers, education and social lives are still developing, and the pandemic has caused substantial disruption to these (Alonzi et al., 2020).

Although the findings of this study provide insight into the impact of the lockdown on PA and wellbeing; there are some limitations. The study was a cross sectional study using a convenience sampling strategy, resulting in a sample largely consisting of females and individuals of a white background. Furthermore, the average SES was high; with no participants ranking their SES as one or two out of ten. This limits the generalisability of the data to the wider population; particularly due to the growing data indicating substantial adverse impacts for individuals from black and ethnic minority groups (Khunti and Pareek, 2020; Laurencin and McClinton, 2020). Furthermore, it is not possible to confirm the direction of cause and effect between the relationships identified; specifically whether people who rated PA as less important engaged in less PA as a result, or whether engaging in less PA led to it being rated as less important. This limitation is also relevant to the wellbeing data; it is not possible to confirm whether low wellbeing led to reduced PA, or whether low PA led to reduced wellbeing. In the follow up from this survey, and as we adapt to living with the coronavirus, examination of long-term changes in PA attitudes, behaviours and wellbeing are needed. It would be interesting to determine whether participants experienced long term changes in their perception of the importance of PA, their PA modes and behaviours; and whether this had an impact on their PA level and wellbeing.

Overall, the findings of this study revealed that PA during lockdown varied by participants perception of the importance of PA with those who rated it as more important doing significantly more of all PA intensities. In addition, individuals with ‘low’ levels of PA and who reported that PA was of less importance during lockdown than it was prior to lockdown, scored worse on all measures of wellbeing. Strategies to overcome the adverse health and wellbeing impacts of the pandemic and its continued influenced on our lives; should focus on increasing PA, perhaps through more widespread promotion of its beneficial effects on wellbeing and use as a coping strategy.

## Research Data

sj-docx-1-hpq-10.1177_1359105321999710 – A cross-sectional study of physical activity behaviour and associations with wellbeing during the UK coronavirus lockdown Click here for additional data file. sj-docx-1-hpq-10.1177_1359105321999710 for A cross-sectional study of physical activity behaviour and associations with wellbeing during the UK coronavirus lockdown by Carly J Wood, Jo Barton and Nina Smyth in Journal of Health Psychology This article is distributed under the terms of the Creative Commons Attribution 4.0 License ( https://creativecommons.org/licenses/by/4.0/) which permits any use, reproduction and distribution of the work without further permission provided the original work is attributed as specified on the SAGE and Open Access pages ( https://us.sagepub.com/en-us/nam/open-access-at-sage).

sj-sps-2-hpq-10.1177_1359105321999710 – A cross-sectional study of physical activity behaviour and associations with wellbeing during the UK coronavirus lockdown Click here for additional data file. sj-sps-2-hpq-10.1177_1359105321999710 for A cross-sectional study of physical activity behaviour and associations with wellbeing during the UK coronavirus lockdown by Carly J Wood, Jo Barton and Nina Smyth in Journal of Health Psychology This article is distributed under the terms of the Creative Commons Attribution 4.0 License ( https://creativecommons.org/licenses/by/4.0/) which permits any use, reproduction and distribution of the work without further permission provided the original work is attributed as specified on the SAGE and Open Access pages ( https://us.sagepub.com/en-us/nam/open-access-at-sage).

sj-spv-3-hpq-10.1177_1359105321999710 – A cross-sectional study of physical activity behaviour and associations with wellbeing during the UK coronavirus lockdown Click here for additional data file. sj-spv-3-hpq-10.1177_1359105321999710 for A cross-sectional study of physical activity behaviour and associations with wellbeing during the UK coronavirus lockdown by Carly J Wood, Jo Barton and Nina Smyth in Journal of Health Psychology This article is distributed under the terms of the Creative Commons Attribution 4.0 License ( https://creativecommons.org/licenses/by/4.0/) which permits any use, reproduction and distribution of the work without further permission provided the original work is attributed as specified on the SAGE and Open Access pages ( https://us.sagepub.com/en-us/nam/open-access-at-sage).
