# Supplementary material for: A cross-sectional study of physical activity behaviour and associations with wellbeing during the UK coronavirus lockdown
Source: J Health Psychol. 2021 Mar 3;27(6):1432–44. doi: 10.1177/1359105321999710 (PMC9036158; doi:10.1177/1359105321999710)
Supplement: sj-docx-1-hpq-10.1177_1359105321999710 – A cross-sectional study of physical activity behaviour and associations with wellbeing during the UK coronavirus lockdown [file sj-docx-1-hpq-10.1177_1359105321999710.docx]

**Explanatory Memo for Data**

Alongside this explanatory memo the following are enclosed:

1. The syntax file derived directly from the analysis. This syntax file contains the codes for all analysis presented in the manuscript;
2. The output file which contains the results of the analysis conducted in SPSS. The output is presented in the order in which the results are presented in the results section of the manuscript.
